# Effect of motivational behavioral change–integrated rehabilitation nursing on recovery and prognosis in patients with hypertension and diabetic foot ulcers

**DOI:** 10.1097/MD.0000000000049643

**Published:** 2026-07-03

**Authors:** Haijuan Yang, Dandan Xiao, Ya Su, Qianbo Zhong, Huifen Wu, Zhiping Huang

**Affiliations:** a Outpatient Department, Hainan Provincial Geriatric Hospital, Haikou City, Hainan Province, China; b Rehabilitation Medicine Department, Hainan Provincial Geriatric Hospital, Haikou City, Hainan Province, China; c Nursing Department, Hainan Provincial Geriatric Hospitalt, Haikou City, Hainan Province, China; d Rehabilitation Medicine Department, Haikou Third People’s Hospital, Hainan Province, China; e Endocrinology Department, Hainan Provincial Geriatric Hospital, Haikou City, Hainan Province, China; f Geriatric Medical Center, Hainan Provincial Geriatric Hospital, Haikou City, Hainan Province, China.

**Keywords:** diabetic foot, hypertension, motivational behavior transformation, rehabilitation nursing

## Abstract

A retrospective analysis was conducted on 72 patients with hypertension complicated by diabetic foot ulcers who were treated in our hospital between January 2020 and February 2023. According to the nursing approach documented in the medical records, patients were assigned to a motivation-oriented behavioral change–based rehabilitation nursing group (n = 36) or a routine nursing group (n = 36). Outcomes included systolic and diastolic blood pressure, fasting and 2-hour postprandial blood glucose, ulcer healing rates at 7, 14, and 28 days, foot function scores, overall clinical effectiveness, self-management ability, treatment adherence, disease knowledge mastery, and diabetes-specific quality of life assessed by the Diabetes-Specific Quality of Life Scale. Baseline characteristics were comparable between groups. After nursing, the rehabilitation nursing group demonstrated lower blood pressure and blood glucose levels than the routine group, with statistically significant between-group differences. Ulcer healing rates were similar at day 7, but were higher in the rehabilitation nursing group at days 14 and 28. Foot function scores improved in both groups, with greater improvement in the rehabilitation nursing group. The total effective rate of foot recovery was higher in the rehabilitation nursing group. In addition, self-management ability, adherence, and knowledge mastery increased after nursing, and post-intervention quality of life scores were lower (indicating better quality of life) in the rehabilitation nursing group. These findings suggest that motivation-oriented behavioral change integrated into rehabilitation nursing is associated with improved short-term clinical and patient-reported outcomes in this population.

## 1. Introduction

In recent years, the incidence of diabetes and hypertension in China has been increasing, making them common chronic diseases in clinical practice among the middle-aged and elderly population. Prolonged hypertension can cause damage to multiple target organs in the body and increase the likelihood of complications.^[1]^ Diabetic foot, as a severe complication of diabetes with a high disability rate, significantly worsens the condition when combined with hypertension. It also complicates clinical treatment and poses risks to overall health.^[2]^ Hypertension and diabetic foot ulcers are significant health concerns that contribute to substantial morbidity and mortality rates worldwide. Therefore, comprehensive management approaches that go beyond medical interventions alone are necessary. In recent years, there has been an increasing emphasis on the role of motivational behavior change within the field of rehabilitation nursing as a potential catalyst for improved patient outcomes.

To address this issue, in addition to conventional treatment, appropriate nursing interventions should be selected to help accelerate the patient’s recovery process. Currently, conventional nursing, which primarily focuses on disease control through dietary adjustments and monitoring of condition changes, is commonly used in clinical practice. However, it lacks sufficient attention to the psychological changes of patients and yields poor prognostic outcomes.^[3]^ An analysis has revealed that rehabilitation nursing based on motivation and behavior transformation has shown significant effects. As a new nursing model, it primarily stimulates patients’ motivation for rehabilitation training and transforms that motivation into behavioral interventions. This approach not only controls blood pressure but also promotes wound ulcer recovery, encourages patients to actively cooperate with nursing care, and provides support for better prognostic outcomes.^[4,5]^

Motivational behavior change is a multidimensional concept encompassing various psychological, social, and environmental factors that influence individuals to adopt and maintain health-promoting behaviors. These behaviors may include adherence to prescribed medication regimens, regular physical activity, dietary modifications, and self-care practices. Incorporating motivational behavior change strategies into rehabilitation nursing practices could potentially enhance patient engagement, empower individuals to take an active role in their own care, and ultimately improve recovery and prognosis.

To further investigate the impact of rehabilitation nursing based on motivation and behavior transformation on the recovery and prognosis of patients with hypertension and diabetic foot, our hospital conducted a grouped study involving 72 cases of such patients. The aim of this manuscript is to comprehensively examine the influence of motivational behavior change within rehabilitation nursing on the recovery and prognosis of patients with hypertension and diabetic foot ulcers. By exploring the existing literature, identifying relevant theoretical frameworks, and examining empirical evidence, this study endeavors to shed light on the potential benefits, challenges, and strategies associated with integrating motivational behavior change interventions into the nursing care of these individuals. The investigation of the influence of motivational behavior change within rehabilitation nursing on the recovery and prognosis of individuals with hypertension and diabetic foot ulcers aims to contribute to the growing body of knowledge and inform evidence-based care practices in this important field.

## 2. Materials and methods

### 2.1. Inclusion and exclusion criteria

This study was approved by the Ethics Committee of Hainan Provincial Geriatric Hospital. The inclusion criteria for this study were defined as follows: patients and their relatives who provided signed informed consent forms; diagnosis in accordance with the “National Guidelines for the Prevention and Control of Primary Hypertension”^[6]^ and the “Expert Consensus on Screening and Prevention of Diabetic Foot at the Primary Level”^[7]^; high compliance and complete medical records. On the other hand, the study employed exclusion criteria that consisted of the following: lactating or pregnant women; presence of neurological disorders, hematological diseases, hepatic or renal insufficiency, mental disorders, or sensory impairments; diabetic ketoacidosis or other complications; a history of amputation before the study.

### 2.2. General data

A total of 72 patients with a confirmed diagnosis of hypertension complicated by diabetic foot who were admitted to our hospital between January 2020 and February 2023 were retrospectively identified. Based on the nursing program documented in the medical records, patients were categorized into a motivation-oriented behavioral change–based rehabilitation nursing group (n = 36) and a routine nursing group (n = 36). Baseline demographic and clinical characteristics were comparable between the two groups, with no statistically significant differences observed (*P* > .05).

### 2.3. Project design

Control Group: the control group received routine nursing care, which included regular dressing changes for foot ulcerations, strict management of blood glucose and blood pressure according to medical instructions, education on disease-related knowledge, self-management techniques, exercise methods, and dietary restrictions. Psychological counseling was provided as needed.

Rehabilitation nursing group: patients in the rehabilitation nursing group received motivation-oriented, behavioral change–based rehabilitation nursing, which consisted of the following components.

Motivational intervention: before nursing implementation, nurses comprehensively assessed patients’ disease status, psychological condition, rehabilitation needs, and level of disease-related knowledge. Through active communication, encouragement, and motivational interviewing techniques, patients were guided to express their concerns and expectations. The adverse consequences of poorly controlled hypertension and diabetic foot ulcers were explained, and successful rehabilitation cases were shared to strengthen patients’ confidence and motivation for recovery.

Health education and behavioral intervention: behavioral interventions were conducted, including regular health knowledge lectures delivered by experts. A WeChat group was established to facilitate timely communication between healthcare providers and patients. Topics covered causes, mechanisms, treatment methods, precautions, and measures for preventing complications of hypertension and diabetic foot. Individualized rehabilitation plans were developed based on each patient’s specific circumstances.

Medication rehabilitation guidance: in addition to conventional glycemic control medication, immediate debridement intervention was undertaken for diabetic foot ulcers. Appropriate sterile gauze was selected, and medication was incorporated into oil-infused gauze. After high-pressure sterilization, the affected area was properly dressed, and regular gauze replacement was ensured. Patients and their families were educated about medication usage and relevant precautions.

Behavioral rehabilitation nursing: various forms and approaches were employed to provide health education. Short videos were utilized to inform patients about the importance of daily foot care, close monitoring of blood glucose levels, and guidance on diet and exercise. Individualized dietary plans were developed in collaboration with a nutritionist. Cognitive assessments were conducted using relevant questionnaires to evaluate patients’ understanding, and personalized health education was provided.

Psychological rehabilitation nursing: various forms and approaches were employed to provide health education. Short videos were utilized to inform patients about the importance of daily foot care, close monitoring of blood glucose levels, and guidance on diet and exercise. Individualized dietary plans were developed in collaboration with a nutritionist. Cognitive assessments were conducted using relevant questionnaires to evaluate patients’ understanding, and personalized health education was provided.

Wound rehabilitation nursing: for the management of diabetic foot ulcer wounds, strict adherence to aseptic principles was observed during nursing procedures, focusing specifically on the affected area.

Dietary planning: Patients were encouraged to consume ample fresh fruits and vegetables while avoiding pickled foods, high-cholesterol, high-sugar, and high-fat foods. The relationship between diet and diabetes/hypertension was explained.

Exercise rehabilitation nursing: suitable exercise modalities were selected based on the patient’s condition and physical status. Personalized exercise plans were formulated, including leg elevation and ankle pump exercises. Exercise duration and intensity were gradually increased following the principle of progression to reduce the occurrence of complications such as lower limb deep vein thrombosis, joint stiffness, and muscle atrophy.

### 2.4. Observation target

Blood glucose and blood pressure levels were measured before and after nursing interventions in both groups. The ulcer healing rate was calculated at 7, 14, and 28 days after nursing interventions using the formula: [(pre-nursing ulcer area − post-nursing ulcer area)/pre-nursing ulcer area] × 100.00%.^[8]^ Foot function was assessed by examining the condition of the feet in both groups and scoring the changes in symptoms such as numbness, dorsalis pedis pulse, and pain. Scores ranged from 0 to 12, with lower scores indicating better foot function.^[9]^ A comparison was conducted between the 2 groups to determine the effects of foot healing. The healing outcomes were categorized as follows: “cured” for complete healing of the limb’s ulcerated area, “significant improvement” for healing of the limb’s ulcerated area by more than two-thirds, “effective” for healing of the limb’s ulcerated area between one-fourth and two-thirds, and “ineffective” for healing of the limb’s ulcerated area less than one-fourth or worsening of the condition.^[10]^ The total effective rate was calculated as (cured + significant improvement + effective)/total number of cases × 100.00%. Self-management ability, compliance, and knowledge proficiency were evaluated before and after nursing interventions. Diabetes self-management ability was assessed using eleven items, each scored on a scale of 1 to 7, with a maximum score of 77, where a higher score indicated stronger self-management ability.^[11]^ Compliance was scored on a scale of 1 to 10, with higher scores indicating better adherence to nursing care.^[12]^ Knowledge proficiency was scored on a scale of 1 to 10, with higher scores denoting a greater understanding of the disease.^[13]^ Quality of life was assessed using the Diabetes-Specific Quality of Life Scale (DSQL).^[14]^ The scale consisted of 25 items encompassing 3 dimensions: physiological function, psychological well-being, and social relationships. Each item was scored from 1 to 5, with lower scores indicating a higher quality of life.

### 2.5. Statistical treatment

The full text data was calculated using the Statistical Package for the Social Sciences (SPSS) 20.0 (IBM Corp.) system. Measurement data was presented as ±s, and the *T*-test was selected. Percentage was used for count data, for which the χ2 test was selected, and *P* < .05 was considered a statistical difference.

## 3. Result

### 3.1. Comparison of general information between the 2 hroups

As indicated in Table 1, there were no significant differences observed between the rehabilitation nursing group and the standard group regarding gender (20 males/16 females vs 18 males/18 females, χ^2^ = 0.223, *P* = .637), age (70.26 ± 1.85 years vs 70.48 ± 1.62 years, *t* = 0.537, *P* = .593), disease duration (4.35 ± 1.02 years vs 4.70 ± 1.11 years, *t* = 1.393, *P* = .168), and diabetes Wagner classification (17 stage I/13 stage II/6 stage III vs 16 stage I/15 stage II/5 stage III, χ^2^ = 0.264, *P* = .876).

**Table 1:** Comparison of general information between the 2 groups.

Group	Male/female (cases)	Age (yr)	Disease duration (yr)	Diabetes wagner classification (stage I/stage II/stage III)
Rehabilitation Nursing Group (n = 36)	20/16	70.26 ± 1.85	4.35 ± 1.02	17/13/6
Standard Group (n = 36)	18/18	70.48 ± 1.62	4.70 ± 1.11	16/15/5
χ^*2*^/*t*	0.223	0.537	1.393	0.264
*P*	.637	.593	.168	.876

### 3.2. Changes in blood glucose and blood pressure between the 2 groups

As presented in Table 2, no significant differences were observed in pre-nursing systolic blood pressure (163.47 ± 7.21 mm Hg vs 163.68 ± 7.04 mm Hg, *t* = 0.125, *P* = .901), diastolic blood pressure (115.78 ± 6.20 mm Hg vs 115.46 ± 6.95 mm Hg, *t* = 0.206, *P* = .837), fasting blood glucose (11.57 ± 3.85 mmol/L vs 12.02 ± 3.96 mmol/L, *t* = 0.489, *P* = .626), and postprandial 2-hour blood glucose (15.47 ± 6.32 mmol/L vs 15.81 ± 6.09 mmol/L, *t* = 0.232, *P* = .817) between the 2 groups. However, following nursing interventions, the rehabilitation nursing group demonstrated significantly lower systolic blood pressure (102.01 ± 4.38 mm Hg vs 123.56 ± 5.91 mm Hg, *t* = 17.577, *P*<.001), diastolic blood pressure (80.69 ± 3.48 mm Hg vs 95.11 ± 4.07 mm Hg, *t* = 16.157, *P*<.001), fasting blood glucose (7.92 ± 1.64 mmol/L vs 9.55 ± 2.01 mmol/L, *t* = 3.770, *P*<.001), and postprandial 2-hour blood glucose (9.05 ± 2.14 mmol/L vs 11.78 ± 3.09 mmol/L, *t* = 4.358, *P*<.001) compared to the standard group. These findings suggest that motivation-based behavioral transformation nursing significantly enhances blood pressure and blood glucose measures among patients with hypertensive diabetic foot.

**Table 2:** Changes in blood glucose and blood pressure between the 2 groups ($\bar{x}\pm \;S$)_._

Group	Systolic blood pressure (mm Hg)		Diastolic blood pressure (mm Hg)		Fasting blood glucose (mmol/L)		Postprandial 2-hour blood glucose (mmol/L)	
	Pre-Nursing	Post-Nursing	Pre-Nursing	Post-Nursing	Pre-Nursing	Post-Nursing	Pre-Nursing	Post-Nursing
Rehabilitation Nursing Group (n = 36)	163.47 ± 7.21	102.01 ± 4.38	115.78 ± 6.20	80.69 ± 3.48	11.57 ± 3.85	7.92 ± 1.64	15.47 ± 6.32	9.05 ± 2.14
Standard Group (n = 36)	163.68 ± 7.04	123.56 ± 5.91	115.46 ± 6.95	95.11 ± 4.07	12.02 ± 3.96	9.55 ± 2.01	15.81 ± 6.09	11.78 ± 3.09
*t*	0.125	17.577	0.206	16.157	0.489	3.770	0.232	4.358
*P*	.901	<.001	.837	<.001	.626	<.001	.817	<.001

### 3.3. Wound ulcer healing rate between the 2 groups

As depicted in Table 3, there was no significant difference observed in the wound ulcer healing rate between the 2 groups after 7 days of nursing (7.78 ± 1.52% vs 7.51 ± 1.46%, *t* = 0.768, *P* = .445). However, at 14 days (39.67 ± 5.21% vs 26.84 ± 4.06%, *t* = 11.655, *P*<.001) and 28 days (52.08 ± 6.37% vs 44.67 ± 5.19%, *t* = 5.411, *P*<.001), the rehabilitation nursing group demonstrated a higher wound ulcer healing rate compared to the standard group. These findings indicate that motivation-based behavioral transformation nursing significantly enhances the wound ulcer healing rate among patients with hypertensive diabetic foot.

**Table 3:** Wound ulcer healing rate between the 2 groups ($\bar{x}\pm \;S$, %).

Group	7 d after nursing	14 d after nursing	28 d after nursing
Rehabilitation Nursing Group (n = 36)	7.78 ± 1.52	39.67 ± 5.21	52.08 ± 6.37
Standard Group (n = 36)	7.51 ± 1.46	26.84 ± 4.06	44.67 ± 5.19
*t*	0.768	11.655	5.411
*P*	.445	<.001	<.001

### 3.4. Comparison of foot function between the 2 groups

As presented in Table 4, there was no significant difference observed in the foot function scores between the 2 groups prior to nursing (9.68 ± 1.02 vs 9.64 ± 1.08, *t* = 0.120, *P* = .905). However, following 14 days (3.31 ± 0.24 vs 4.58 ± 0.57, *t* = 10.355, *P*<.001) and 28 days (1.85 ± 0.12 vs 2.68 ± 0.29, *t* = 13.556, *P*<.001) of nursing, the rehabilitation nursing group exhibited significantly lower foot function scores compared to the standard group. These findings suggest that motivation-based behavioral transformation nursing significantly enhances foot function among patients with hypertensive diabetic foot.

**Table 4:** Comparison of foot function between the 2 groups ($\bar{x}\pm \;S$, score).

Group	n	Foot function		
		7 d after nursing	14 d after nursing	28 d after nursing
Rehabilitation Nursing Group	36	9.68 ± 1.02	3.31 ± 0.24	1.85 ± 0.12
Standard Group	36	9.64 ± 1.08	4.58 ± 0.57	2.68 ± 0.29
*t*	–	0.120	10.355	13.556
*P*	–	.905	<.001	<.001

### 3.5. Comparison of foot recovery effects between the 2 groups

As depicted in Table 5, the rehabilitation nursing group exhibited 10 cases of recovery, 20 cases of significant improvement, 5 cases of improvement, and 1 case of ineffectiveness. On the other hand, the standard group experienced 6 cases of recovery, 15 cases of significant improvement, 8 cases of improvement, and 7 cases of ineffectiveness. The total effective rate of foot recovery in the rehabilitation nursing group (97.22% vs 80.56%, χ^2^ = 5.063, *P* = .024) surpassed that of the standard group. These findings indicate that motivation-based behavioral transformation rehabilitation nursing leads to superior foot function recovery.

**Table 5:** Comparison of foot recovery effects between the 2 groups (n, %).

Group	Recovery	Significant improvement	Improvement	Ineffectiveness	Total effective rate (%)
Rehabilitation Nursing Group (n = 36)	10	20	5	1	35 (97.22)
Standard Group (n = 36)	6	15	8	7	29 (80.56)
*χ^2^*	–	–	–	–	5.063
*P*	–	–	–	–	.024

### 3.6. Changes in self-management ability, compliance, and knowledge mastery in the 2 groups

As presented in Table 6, there were no significant differences observed in self-management ability (35.21 ± 6.78 vs 35.64 ± 6.22, *t* = 0.280, *P* = .780), compliance (4.69 ± 1.56 vs 4.30 ± 1.33, *t* = 1.141, *P* = .258), and knowledge mastery (5.02 ± 1.63 vs 5.44 ± 1.70, *t* = 1.070, *P* = .288) between the 2 groups prior to nursing care. However, following care, the rehabilitation nursing group displayed higher scores in self-management ability (63.26 ± 1.45 vs 57.55 ± 2.63, *t* = 11.408, *P*<.001), compliance (8.19 ± 0.64 vs 7.56 ± 0.88, *t* = 3.474, *P*<.001), and knowledge mastery (8.57 ± 0.42 vs 7.90 ± 0.81, *t* = 4.406, *P*<.001) compared to the standard group. These findings indicate that motivation-based behavioral transformation rehabilitation nursing significantly enhances self-management ability, compliance, and knowledge mastery among patients with hypertension and diabetes-related foot disorders.

**Table 6:** Changes in self-management ability, compliance, and knowledge mastery in the 2 groups ($\bar{x}\pm \;S$, scores).

Group	Self-management ability		Compliance		Knowledge mastery	
	Before care	After care	Before care	After care	Before care	After care
Rehabilitation Nursing Group (n = 36)	35.21 ± 6.78	63.26 ± 1.45	4.69 ± 1.56	8.19 ± 0.64	5.02 ± 1.63	8.57 ± 0.42
Standard Group (n = 36)	35.64 ± 6.22	57.55 ± 2.63	4.30 ± 1.33	7.56 ± 0.88	5.44 ± 1.70	7.90 ± 0.81
*t*	0.280	11.408	1.141	3.474	1.070	4.406
*P*	.780	<.001	.258	<.001	.288	<.001

### 3.7. Comparison of DSQL scores between the 2 groups

As depicted in Table 7, there were no significant differences observed in physiological function (30.21 ± 1.61 vs 29.97 ± 1.54, *t* = 0.754, *P* = .453), psychological dimension (16.92 ± 2.24 vs 16.51 ± 2.28, *t* = 0.898, *P* = .371), and social relationships (7.51 ± 0.93 vs 7.48 ± 0.89, *t* = 0.163, *P* = .871) scores between the 2 groups prior to nursing care. However, following care, the rehabilitation nursing group exhibited significantly lower scores in physiological function (14.51 ± 1.02 vs 23.14 ± 2.69, *t* = 20.998, *P*<.001), psychological dimension (7.73 ± 1.05 vs 13.15 ± 1.11, *t* = 24.831, *P*<.001), and social relationships (3.35 ± 0.51 vs 5.55 ± 1.16, *t* = 12.153, *P*<.001) compared to the standard group. These findings suggest that motivation-based behavioral transformation rehabilitation nursing can substantially improve the quality of life in hypertensive patients with diabetes-related foot disorders (Table 7).

**Table 7:** Comparison of DSQL scores between the 2 groups ($\bar{x}\pm \;S$, scores).

Group	Physiological function		Psychological dimension		Social relationships	
	Before care	After care	Before care	After care	Before care	After care
Rehabilitation Nursing Group (n = 36)	30.21 ± 1.61	14.51 ± 1.02	16.92 ± 2.24	7.73 ± 1.05	7.51 ± 0.93	3.35 ± 0.51
Standard Group (n = 36)	29.97 ± 1.54	23.14 ± 2.69	16.51 ± 2.28	13.15 ± 1.11	7.48 ± 0.89	5.55 ± 1.16
*t*	0.754	20.998	0.898	24.831	0.163	12.153
*P*	.453	<.001	.371	<.001	.871	<.001

DSQL = Diabetes-Specific Quality of Life Scale.

## 4. Discussion

Hypertension is characterized by a sustained increase in blood pressure, posing significant risks to patients’ overall health. Diabetic foot, a common complication of diabetes, is often caused by vascular or macrovascular disease and is highly prone to infection, making it challenging to treat.^[15]^ Furthermore, research indicates that hypertensive patients in a hyperglycemic state have an optimal environment for bacterial growth due to reduced phagocytic ability of white blood cells, thereby increasing the risk of infection.^[16]^ Given these circumstances, it is crucial for clinicians to go beyond routine treatments and actively choose effective nursing interventions to expedite wound healing, achieve prompt control of blood pressure and blood sugar, and enhance patient awareness.^[17]^

Traditionally, clinical practice relied on conventional nursing interventions that lacked specificity and functioned more as one-way health education to patients, leaving them with an incomplete understanding of disease-related knowledge.^[18]^ However, as clinical analysis progressed, it became evident that rehabilitative nursing based on behavior change theory was more effective. This approach transformed motivation into rehabilitative nursing and represented a scientifically sound, systematic, and standardized nursing model. By tailoring nursing measures to individual patient needs, stimulating motivation for rehabilitation, and reducing non-health behaviors and high-risk behaviors, this nursing approach has been successfully applied to various diseases, such as improving dialysis-related knowledge and promoting patient participation in treatment and recovery.^[19,20]^ Nevertheless, the application of this nursing model in hypertensive patients with diabetic foot has not been previously reported. This study aims to fill this gap, and the results reveal that the rehabilitation nursing group demonstrated better blood pressure and blood glucose indicators than the conventional group (*P* < .05). These positive outcomes were attributed to several factors. Specialized physician guidance allowed for personalized medication adjustments, resulting in precise hypertension and hyperglycemia control. Collaboration with nutritionists in developing personalized dietary plans assisted in blood sugar and blood pressure control by avoiding high-sugar, high-salt, and high-fat foods. Improved patient awareness and willingness to engage in self-management, facilitated through effective communication and health education, led to better control of blood sugar and blood pressure. Additionally, moderate exercise contributed to improved insulin sensitivity and lower blood glucose levels, while also proving effective in lowering blood pressure. Psychological counseling and stress management techniques played a role in reducing blood pressure and blood glucose levels. Enhancing patients’ disease awareness and self-management skills encouraged active participation in their own treatment and management, further optimizing blood sugar and blood pressure control. The utilization of modern communication tools, such as short videos and WeChat groups, facilitated efficient delivery of health information and follow-up on patient conditions, enhancing education and management.^[21]^ Furthermore, this study observed a higher rate of ulcerated wound recovery in the foot in the rehabilitation group after 14 and 28 days of nursing compared to the conventional group. The foot function scores were also lower in the rehabilitation group. Several reasons account for these findings. Effective medication treatment, including antibiotics or other ointments, controlled infection and promoted wound healing. Individualized rehabilitation plans tailored to each patient’s needs enabled timely adjustments and expedited wound healing. Strict adherence to aseptic principles and local treatment methods reduced the risk of infection, accelerating wound healing. Adequate nutritional intake, particularly high-protein foods, supported the body’s self-repair mechanisms and aided in wound healing. Psychological rehabilitation care positively influenced the body’s response to treatment, potentially accelerating wound healing.^[22]^ Appropriate exercise programs, such as leg elevation and ankle pumping exercises, improved foot function and reduced the risk of complications like lower limb venous thrombosis, joint stiffness, and muscle atrophy. Behavioral rehabilitation care emphasized educating patients on proper foot care in daily life, reducing foot problems caused by improper care. Knowledge dissemination and education provided patients with the necessary information to enhance foot health and function. Social and psychological support, including support groups and a positive psychological state, assisted patients in better managing their symptoms, promoting foot function and overall well-being.^[23]^

Additionally, the study found that the rehabilitation group scored lower in physiological function, psychological dimension, and social relationships compared to the conventional group (*P* < .05). These results have diverse origins. Physiologically, rehabilitation care improved hypertension and blood glucose levels, accelerated wound healing, and reduced the risk of complications through rational medication use, exercise intervention, and wound care. These interventions collectively enhanced patients’ physical comfort and self-management capabilities. In terms of psychological support, approaches such as support groups, psychological counseling, and health education increased disease awareness and self-management abilities, alleviating psychological stress and promoting mental well-being.^[24]^ Furthermore, knowledge education delivered through various mediums empowered patients with a better understanding of their condition and more effective self-management strategies, ultimately improving treatment compliance. Lastly, rehabilitation care encouraged patients to engage in social activities, such as support groups, providing not only social support but also instilling confidence through the success stories of others.^[25]^

In summary, during the rehabilitation period of behavior transformation, motivational interventions were implemented to empower patients with disease-related knowledge, stabilize emotions, eliminate negative mindsets, convey the importance of self-management, and enhance confidence in overcoming the disease. Subsequently, behavioral interventions incorporated health education into various aspects of patients’ daily lives, guiding them towards adopting a healthy lifestyle, cultivating beneficial habits, and providing instructions on blood pressure monitoring and foot care methods. Tailored exercise and dietary plans, based on individual patients’ conditions and physical states, effectively lowered blood pressure and blood glucose levels, accelerated wound healing, enhanced self-management abilities, improved compliance, and ultimately elevated patients’ self-care capabilities and overall quality of life. These interventions effectively lowered blood pressure and blood glucose levels, accelerated wound healing, enhanced self-management abilities, improved compliance, and ultimately elevated patients’ self-care capabilities and overall quality of life.

## Author contributions

**Conceptualization:** Haijuan Yang, Dandan Xiao, Ya Su, Qianbo Zhong, Huifen Wu, Zhiping Huang.

**Data curation:** Haijuan Yang, Dandan Xiao, Ya Su, Qianbo Zhong, Huifen Wu, Zhiping Huang.

**Formal analysis:** Haijuan Yang, Dandan Xiao, Ya Su, Qianbo Zhong, Huifen Wu, Zhiping Huang.

**Funding acquisition:** Haijuan Yang, Dandan Xiao, Ya Su, Qianbo Zhong, Huifen Wu, Zhiping Huang.

**Investigation:** Haijuan Yang, Zhiping Huang.

**Writing – original draft:** Zhiping Huang.

**Writing – review & editing:** Zhiping Huang.
